# Work arrangements, food-related time scarcity, and meal delivery use: a cross-sectional analysis of the 2024 Belgian Meal Delivery Survey

**DOI:** 10.1093/eurpub/ckag144

**Published:** 2026-07-30

**Authors:** Laura Helena Oostenbach, Maartje P Poelman, Lukar Thornton

**Affiliations:** Department of Marketing, Faculty of Business and Economics, University of Antwerp, Antwerp, Belgium; Department of Social Sciences, Chair Group Consumption & Healthy Lifestyles, Wageningen University & Research, Wageningen, The Netherlands; Department of Marketing, Faculty of Business and Economics, University of Antwerp, Antwerp, Belgium

## Abstract

Demanding work arrangements may contribute to food-related time scarcity and influence food practices, including meal delivery use. This study investigated links between work arrangements, time scarcity in food shopping and cooking, and meal delivery use and motivations. Complete-case (94%) analysis included 674 paid workers from the 2024 Belgian Meal Delivery Survey. Generalised ordered logit models examined associations between weekly work hours, workweek length, schedule, and onsite workdays with time scarcity and meal delivery use. Amongst users, logistic regression examined associations between work arrangements and reasons for ordering delivery. Models adjusted for socio-demographic and household characteristics. Longer hours were associated with higher odds of lacking time to shop [odds ratio (OR) = 1.03, 95% confidence interval (CI): 1.00, 1.06] and cook (OR = 1.05, 95% CI: 1.02, 1.07). A ≥5-day workweek was associated with higher odds of lacking time to shop (OR = 2.21, 95% CI: 1.09, 4.51) and cook (OR = 3.02, 95% CI: 1.46, 6.27). Non-regular schedules were associated with meal delivery use ≥3/month (OR = 1.75, 95% CI: 1.09, 2.80). More days onsite were linked to higher odds of lacking time to shop (OR = 1.18, 95% CI: 1.01, 1.37). Amongst users, longer hours and ≥5-day workweeks were linked to ordering meals to support work tasks (OR = 1.06, 95% CI: 1.03, 1.09; OR = 2.15, 95% CI: 1.19, 3.91) but not for household, leisure or healthy-activity reasons. Demanding work arrangements were consistently associated with food-related time scarcity but not with more frequent meal delivery use, except for non-regular schedules. When used, meal delivery served to facilitate completing work demands.

## Introduction

Participation in paid labour is an important aspect of everyday adult life. In Belgium, 73% of those aged 20–64 years old were active in the workforce in 2024 [1], with 74% of employees working full-time jobs [2]. Balancing full-time employment with personal responsibilities can be difficult and may lead to a sense that work leaves little time for other parts of life. Work arrangements, such as long hours and non-standard schedules, are linked to stronger feelings of time scarcity [3] and can limit time and energy available for daily activities, including food-related tasks [4, 5]. Workers may rush or shorten activities, multitask, or delegate tasks such as cooking to external services [4, 5]. Previous research has suggested that demanding work arrangements are linked to less time spent grocery shopping and cooking at home [6], and greater reliance on convenience foods [7, 8], with detrimental dietary and health implications [9]. However, much of the existing evidence is from a pre- or during-COVID context [6–8]. Since the pandemic, work arrangements have shifted in terms of where and how people work, with increased remote work potentially altering daily routines and temporal flexibility, and in turn affecting experiences of time scarcity [10]. These work changes have also been paralleled with the rise of ready-to-eat meal delivery services, which has increased accessibility to these services and contributed to the normalisation of meal delivery use [11]. This changed context provides scope for investigation of transformed work arrangements and food-related behaviours amongst the post-COVID workforce.

Working from home has become more common in Belgium and other high-income countries since the pandemic [10, 12]. Whilst 19% of Belgian employees worked from home at least sometimes in 2019, this figure rose to 33% in 2024, mainly amongst highly skilled workers [12]. However, most employees still spend the majority of their workdays onsite [10]. Being onsite for work may impose time demands through, e.g. preparation for a workday away from home and commuting to and from the workplace, which can reduce time and flexibility for food-related tasks. Pre-pandemic research suggested onsite workers spent less time on food-related activities (e.g. grocery shopping, cooking) than those working from home [13]. Research on work location and food-related outcomes remains nonetheless mixed and scarce [14, 15]. As many workers continue to work onsite [10, 12], it is important to understand how onsite workdays impact workers’ experience of time scarcity and food-related behaviours.

Along with these work changes, third-party ready-to-eat meal delivery platforms (e.g. Takeaway.com, Uber Eats) have become increasingly popular [11]. Whilst an estimated 2.1 million people used ready-to-eat meal delivery services in Belgium in 2019, this number increased to 4.0 million in 2024 [11] for a population of less than 12 million [16]. These services offer access to out-of-home meals with minimal time and effort, reducing the need to plan, shop, or prepare food. However, a disproportionate number of meal options offered are energy-dense and nutrient-poor, although heterogeneity exists across cuisines and meal types [17]. This matters from a public health perspective as frequent ordering from meal delivery services may contribute to poorer overall dietary patterns [17]. Despite the growth of these services [11], very little research has explored links between work arrangements and meal delivery services use, with existing studies, conducted pre-COVID, finding no clear association [18, 19].

Seeking time saving has been reported as a general driver of ready-to-eat meal delivery use [20, 21]. However, the purpose of that saved time remains largely unknown. For workers, meal delivery services might be used to redirect time into household chores or personal time to recover from work, or to continue working. Understanding the motivations for using meal delivery services, and how these relate to work arrangements, is important for interpreting patterns of use amongst workers and for informing public health strategies aimed at promoting healthy food behaviours.

From a theoretical perspective, this study draws on the conservation of resources theory and the job demands-resources model [22, 23]. Work arrangements create demands (e.g. time pressure, commuting burden, schedule disruption). These demands can deplete personal resources (especially time and energy). In response, individuals engage in behavioural adaptations to protect remaining resources and prevent further loss [22, 23]. One possible coping strategy may be using meal delivery services to reduce the time and effort required for planning, shopping, and cooking. In that sense, meal delivery services may help workers manage competing work and non-work demands.

This study examines how specific work arrangements (weekly work hours, ≥5-day workweek, work schedule, and days working onsite) shape workers’ experience of food-related time scarcity (lack of time to shop or cook) and the frequency of ready-to-eat meal delivery use. Amongst workers who use meal delivery services, the study further investigates how work arrangements relate to both frequency of use and underlying motivations for ordering meal delivery. No prior research has jointly examined multiple work arrangements together with food-related time scarcity and motivational drivers of meal delivery use in a post-COVID context. Many high-income countries, including Belgium, have seen a rapid growth in meal delivery services and working-from-home arrangements in recent years, reflecting broader shifts in both food environments and work practices [10–12]. We expect that more demanding work arrangements will be associated with greater food-related time scarcity and higher frequency of meal delivery use. Different work arrangements might impact time scarcity and meal delivery use in distinct ways: e.g. long work hours and extended workweeks may directly reduce time available for food-related tasks [6], non-regular schedules may disrupt food-related routines [24, 25], and days onsite may exacerbate time demands through commute and time away from home reducing flexibility around food-related tasks [26]. The findings will advance our understanding of how current work structures relate to food-related time scarcity and meal delivery behaviours amongst working individuals in Belgium, with implications for worker health.

## Methods

### Study design and participants

Data were from 720 participants who completed the cross-sectional online Meal Delivery Survey and reported paid work as their primary activity. The survey was developed as part of the Research Foundation Flanders (FWO) funded project ‘Tracking the growth and impact of the ready-to-eat meal delivery system in Belgium’. Whilst the survey focused on meal delivery usage, it also collected data on food-related time scarcity and more general information such as socio-demographic characteristics and work-related arrangements. Most survey questions were replicated or adapted from various previous surveys (e.g. [27, 28]). The survey was available in English, Dutch, and French. It was administered in Qualtrics and conducted between April and July 2024.

Participants were recruited online through advertising on different social media platforms, including Facebook, Instagram, LinkedIn, and Reddit. Eligible participants had to be aged ≥18 years and live in the Flanders or Brussels region of Belgium. Supplementary File S1 provides a flowchart of participants included in this study. Additional details on survey design and participant recruitment are published elsewhere [29]. Ethics approval was obtained from the University of Antwerp Ethics Committee for the Social Sciences and Humanities (ex_SHW_2023_32_1).

### Exposures

Exposures of this study included (i) usual weekly work hours in paid job(s) (continuous), (ii) workweek length (<5-day workweek vs. ≥5-day workweek), (iii) work schedule [regular daytime schedule vs. non-regular schedule (e.g. night shift, irregular schedule)], and (iv) number of days working onsite. Supplementary File S2 details the corresponding survey questions and response options for all variables relevant to the present analysis. The full survey is available online [30].

### Outcomes

Primary outcomes were (i) no time to shop for food (never or rarely; sometimes; most of the time or always), (ii) no time to prepare and/or cook a meal (hereafter, ‘no time to cook’) (never or rarely, sometimes, most of the time or always), and (iii) meal delivery services use (i.e. ordering ready-to-eat meals delivered to the home, either through third-party meal delivery platforms or directly with restaurants via website or phone) (never, <1/month, 1–2/month, ≥3/month). Secondary outcomes were reasons for ordering meal delivery amongst workers using meal delivery services: (i) easier to finish paid employment tasks (no/yes), (ii) easier to finish household chores (no/yes), (iii) time for healthy activities (no/yes), and (iv) time for leisure activities (no/yes).

### Confounders

Potential confounders included age (years), sex (male; female), household composition (lone adult without children, multiple adults without children in household, household with children aged ≤17 years old), and education (non-university, university).

### Statistical analysis

Separate generalised ordered logit models were fitted to examine associations between each exposure and ordinal outcome (shopping time scarcity, cooking time scarcity, meal delivery use). These models were selected based on Williams [31, 32] who has recommended such models as they address limitations of both traditional ordinal logistic regression and multinomial (polytomous) logistic regression. Ordinal logistic models assume the effect of the exposure is constant across all levels of the outcome, i.e. proportional odds assumption, which is frequently violated in practice. Multinomial logistic models avoid this assumption, but they do not use the ordering of the outcome categories, are far less parsimonious and more difficult to interpret. Generalised ordered logit models keep the ordered structure of the outcome whilst allowing exposure effects to vary across thresholds. If an outcome has K categories, the model estimates K-1 thresholds. For a three-category ordinal outcome, effects can vary across two thresholds: (i) low vs. middle and high and (ii) low and middle vs. high. We would thus estimate two separate effects, i.e. one for each threshold. Importantly, this flexibility makes it possible to detect differences in the magnitude (and direction) of associations across outcome thresholds. For example, an exposure may be associated only with the highest threshold but not with the lower one. This means there would be no effect on moving from the lowest category to higher categories (no effect at threshold 1) but there would be an increase or decrease in the odds of moving from lower categories to the highest category (an effect at threshold 2). This approach can thus help identify more extreme (behavioural) responses. Such patterns could be missed in traditional ordinal logistic regression models [31, 32]. Amongst users of meal delivery services, sub-analyses included generalised ordered logit models examining associations between each exposure and meal delivery services use and logistic models examining associations between exposure and reason for ordering meal delivery.

A complete-case analysis was conducted assuming data were missing completely at random. Characteristics for the complete-case sample appeared to be comparable to the full sample (see Supplementary File S3).

All analyses were conducted using Stata 19.0. The user-written command *gologit2* was used to fit the generalised ordered logit models [32]. All models were adjusted for potential confounders. Additional analyses included work hours and workweek length models adjusting for household income (results not shown).

## Results

Of the 720 participants who completed the survey and reported paid work as their primary activity, 674 (94%) were included in the complete-case analysis [median age: 34 years old; 57% (*n* = 384) female]. Descriptive statistics of exposures, outcomes, and potential confounders are presented in Table 1.

**Table 1.** Descriptive statistics

Variable	Complete case *n* = 674
Weekly work hours, median (25th perc., 75th perc.)	40.0 (37.5, 40.0)
Workweek length	
<5-day workweek	102 (15.1%)
≥5-day workweek	572 (84.9%)
Work schedule	
Regular daytime schedule	568 (84.3%)
Non-regular schedule	106 (15.7%)
Number of days working onsite, median (25th perc., 75th perc.)	3.0 (2.0, 5.0)
No time to shop for food	
Never/rarely	274 (40.7%)
Sometimes	285 (42.3%)
Most of the time/always	115 (17.1%)
No time to cook	
Never/rarely	217 (32.2%)
Sometimes	323 (47.9%)
Most of the time/always	134 (19.9%)
Frequency of meal delivery use	
Never	215 (31.9%)
<1/month	141 (20.9%)
1–2/month	172 (25.5%)
≥3/month	146 (21.7%)
Reason for meal delivery use: easier to finish paid employment tasks (users only, *n* = 459)	191 (41.6%)
Reason for meal delivery use: easier to finish household chores (users only, *n* = 459)	238 (51.9%)
Reason for meal delivery use: time for healthy activities (users only, n = 459)	72 (15.7%)
Reason for meal delivery use: time for leisure activities (users only, *n* = 459)	321 (69.93%)
Age, median (25th perc., 75th perc.)	34 (29, 43)
Sex	
Male	290 (43.0%)
Female	384 (57.0%)
Household composition	
Lone adult without children	185 (27.4%)
Multiple adults without children	293 (43.5%)
Household with children	196 (29.1%)
Education	
Non-university	245 (36.4%)
University	429 (63.6%)

### Associations with weekly work hours

Greater work hours were associated with higher odds of frequently experiencing no time to shop for food (Table 2). More specifically, the odds of experiencing no time to shop at least sometimes (i.e. the lower outcome threshold) increased by 3% per additional hour worked [odds ratio (OR) = 1.03, 95% confidence interval (CI): 1.01, 1.05]. Similarly, the odds of experiencing no time to shop most of the time or always (i.e. the highest outcome threshold) increased by 3% (OR = 1.03, 95% CI: 1.01, 1.06). A similar pattern was observed for no time to prepare or cook a meal. The odds of experiencing no time to cook at least sometimes increased by 3% (OR = 1.03, 95% CI: 1.01, 1.05), and by 5% for the highest outcome threshold (OR = 1.05, 95% CI: 1.02, 1.07). No associations were observed between work hours and meal delivery use (Table 3).

**Table 2.** Separate generalised ordered logit models of food-related time scarcity *N* = 674^a^

	Lowest outcome threshold: never/rarely vs sometimes and most of the time/always		Highest outcome threshold: never/rarely and sometimes vs most of the time/always	
	OR (95% CI)	*P*-value	OR (95% CI)	*P*-value
Work hours (continuous, for each 1-h increase)				
No time to shop	1.03 (1.01; 1.05)	.013	1.03 (1.00; 1.06)	.032
No time to cook	1.03 (1.01; 1.05)	.012	1.05 (1.02; 1.07)	.001
≥5-day workweek (ref. <5-day workweek)				
No time to shop	1.25 (0.80; 1.96)	.336	2.21 (1.09; 4.51)	.028
No time to cook	1.05 (0.65; 1.70)	.832	3.02 (1.46; 6.27)	.003
Non-regular work schedule (ref. regular daytime schedule)				
No time to shop	0.75 (0.49; 1.16)	.194	1.70 (1.00; 2.87)	.049
No time to cook	1.13 (0.72; 1.79)	.595	1.63 (0.99; 2.68)	.055
Days working onsite (continuous, for each 1-day increase)				
No time to shop	1.03 (0.93; 1.14)	.585	1.18 (1.01; 1.37)	.036
No time to cook	1.07 (0.96; 1.19)	.230	1.07 (0.93; 1.22)	.335

a The estimate represents the increase (or decrease) in the OR for being in a higher outcome category at each outcome threshold. For a continuous exposure, it represents change in OR for each 1-unit increase in exposure. For a binary exposure, it represents change in OR compared to the reference exposure category. All models were fitted separately and adjusted for age, sex, household composition, and education. Models of work hours and workweek length also adjusted for work schedule. Additional analyses included models also adjusting for household income, and no major differences in magnitude or direction of effects were observed (results not shown).

OR, odds ratio; 95% CI, 95% confidence interval.

**Table 3.** Separate generalised ordered logit models of ready-to-eat meal delivery use *N* = 674^a^

	Lowest outcome threshold: never vs <1/month, 1–2/month, ≥3/month		Middle outcome threshold: never, <1/month vs 1–2/month, ≥3/month		Highest outcome threshold: never, <1/month, 1–2/month vs ≥3/month	
	OR (95% CI)	*P*-value	OR (95% CI)	*P*-value	OR (95% CI)	*P*-value
Work hours (continuous, for each 1-h increase)						
Meal delivery use	1.01 (0.99; 1.04)	.295	1.00 (0.98; 1.02)	.788	1.00 (0.98; 1.02)	.908
≥5-day workweek (ref. <5-day workweek)						
Meal delivery use	0.99 (0.61; 1.60)	.967	1.02 (0.65; 1.59)	.948	1.24 (0.71; 2.20)	.451
Non-regular work schedule (ref. regular daytime schedule)						
Meal delivery use	1.30 (0.82; 2.08)	.270	1.35 (0.88; 2.06)	.165	1.75 (1.094 2.80)	.020
Days working onsite (continuous, for each 1-day increase)						
Meal delivery use	0.96 (0.86; 1.07)	.433	0.94 (0.84; 1.04)	.229	1.07 (0.94; 1.21)	.318

a See note of Table 2.

Although the estimated effects were small, they change exponentially as weekly work hours accumulate. For example, for each 8-h-per-week increase, the odds of experiencing no time to cook most of the time or always increased by 48% (1.05^8 = 1.48). Predicted probabilities are reported in Supplementary File S4.

### Associations with ≥5-day workweek

Workers with a ≥5-day workweek had higher odds of experiencing no time to shop for food (OR = 2.21, 95% CI: 1.09, 4.51) or no time to prepare or cook (OR = 3.02, 95% CI: 1.46, 6.27) most of the time or always (i.e. at the highest outcome threshold) compared to those working a <5-day workweek (Table 2). No associations were observed for the lower threshold of no time to shop and no time to cook outcomes or for meal delivery use (Table 3). Predicted probabilities are reported in Supplementary File S4.

### Associations with work schedule

Compared to those working regular daytime schedules, workers with non-regular schedules had higher odds of experiencing no time to shop most of the time or always (highest outcome threshold), with an OR of 1.70 (95% CI: 1.00, 2.87) (Table 2). No association was observed for the lower threshold of no time to shop. For no time to cook, the odds were also higher amongst those working non-regular schedules for the highest threshold, although the CI contained the null (OR = 1.63, 95% CI: 0.99, 2.68). When it comes to meal delivery services, workers with non-regular schedules had higher odds of using those services at least three times per month (i.e. at the highest outcome threshold) (OR = 1.75, 95% CI: 1.09, 2.80). No associations were observed for the lower usage thresholds (Table 3). Predicted probabilities are reported in Supplementary File S4.

### Associations with days working onsite

More days working onsite were associated with no time to shop at the higher threshold (Table 2). For each additional day onsite, the odds of experience no time to shop most of the time or always increased by 18% (OR = 1.18, 95% CI: 1.01, 1.37), but no association was observed for the lower threshold. Days onsite were not associated with having no time to cook (Table 2) or meal delivery services use (Table 3). Predicted probabilities are reported in Supplementary File S4.

### Sub-analyses amongst workers using meal delivery services (*n* = 459)

Sub-analyses amongst workers who used meal delivery services showed associations between work arrangements and frequency of meal delivery use that were consistent with the main analyses. No associations were observed, except for non-regular schedules that were linked to higher odds of using meal delivery services at least three times a month (Supplementary File S5). When it comes to reasons for meal delivery services use, greater work hours were associated with higher odds of reporting that meal delivery is used to make it easier to finish paid employment tasks (OR = 1.06, 95% CI: 1.03, 1.09) (Table 4). No associations were observed with reporting that meal delivery is used to make it easier to finish household chores or give more time for healthy or leisure activities. Similarly, workers with a ≥5-day workweek had higher odds of using meal delivery to make it easier to finish paid employment tasks (OR = 2.15, 95% CI: 1.19, 3.91), but no associations were observed with the other reasons. Work schedule (regular daytime vs. non-regular) and days working onsite were not associated with any of the listed reasons for using meal delivery (Table 4).

**Table 4.** Separate logistic models of reasons for ordering ready-to-eat meal delivery services *N* = 459^a^

	OR (95% CI)	*P*-value
Work hours (continuous, for each 1-h increase)		
Easier to finish paid employment tasks	1.06 (1.03; 1.09)	<.001
Easier to finish household chores	1.00 (0.98; 1.03)	.761
Time for healthy activities	1.02 (0.98; 1.05)	.374
Time for leisure activities	1.01 (0.98; 1.04)	.445
≥5-day workweek (ref. <5-day workweek)		
Easier to finish paid employment tasks	2.15 (1.19; 3.91)	.012
Easier to finish household chores	0.92 (0.53; 1.59)	.759
Time for healthy activities	0.79 (0.37; 1.69)	.538
Time for leisure activities	0.95 (0.53; 1.70)	.861
Non-regular work schedule (ref. regular daytime schedule)		
Easier to finish paid employment tasks	1.47 (0.89; 2.45)	.136
Easier to finish household chores	0.95 (0.58; 1.57)	.844
Time for healthy activities	0.69 (0.32; 1.47)	.337
Time for leisure activities	0.71 (0.42; 1.21)	.208
Days working onsite (continuous, for each 1-day increase)		
Easier to finish paid employment tasks	1.04 (0.92; 1.19)	.525
Easier to finish household chores	0.97 (0.86; 1.10)	.651
Time for healthy activities	0.88 (0.74; 1.04)	.134
Time for leisure activities	0.97 (0.84; 1.11)	.610

a The estimate represents the increase (or decrease) in the OR of reporting that specific reason as a reason for ordering meal delivery. See note of Table 2.

## Discussion

This study investigated how work arrangements relate to food-related time scarcity and meal delivery service use amongst workers in Belgium. More demanding arrangements (longer work hours, ≥5-day workweek, non-regular schedules, more onsite days) were associated with greater food-related time scarcity, especially at the highest threshold (most of the time/always), and most consistently for lack of time to shop. However, these same work arrangements were not associated with how often workers ordered meal delivery. The exception was for non-regular schedules which were linked to higher odds of using meal delivery services at least three times a month. Amongst those who used meal delivery services, we also explored associations between work arrangements and reasons for ordering meal delivery. We found longer work hours and ≥5-day workweeks were related to using meal delivery services to support paid employment tasks. These arrangements were not associated with ordering meals to ease household chores or create personal time.

Demanding work arrangements are important contributors to feeling time scarce [3, 4]. Our study extends prior research by suggesting these arrangements are also linked to food-related time scarcity, specifically lacking time to shop for food or cook at home. Importantly, these associations were most pronounced at the highest threshold of time scarcity (reporting lacking time most of the time or always). This aligns with previous findings that workers with longer hours tend to cook less frequently than those with greater leisure time availability [6]. Given our finding linking days worked onsite and lacking time to shop, working-from-home arrangements may hold the potential to reduce time pressures, especially those related to food shopping practices. However, such arrangements are generally accessible to white-collar workers, whilst blue-collar workers, e.g. machine operators or trade workers, are largely excluded [12]. Policies should be designed and implemented to support blue-collar workers in reducing work-related time demands.

Our findings also raise the importance of workplace-based interventions, particularly for workers who cannot work from home. Improving access to healthy food directly within the workplace may help reduce reliance on less healthy convenience options when time for shopping and cooking is limited. Examples could include the provision of healthy food on worksites (e.g. free fruit and affordable healthy canteen meals) and ensuring access to these options beyond standard daytime hours, such as extending canteen opening hours for shift workers.

Whilst reporting time pressures, workers with longer work hours and workweeks did not necessarily resort to more frequent meal delivery. Although meal delivery is one possible response to time scarcity [20, 21], other factors seem to shape the use of meal delivery. It could be that ordering in may not be the primary or preferred option for workers in the Belgian context, where cooking and eating together are culturally important practices [33]. It is plausible that busy workers may instead adopt alternative strategies to manage food-related tasks, such as stricter planning around food shopping and preparation. Previous research has suggested that workers with greater time demands tend to visit their main supermarket less often [19] and leave longer time intervals between maintenance shopping activities, including grocery shopping [34]. Workers, who lack time to shop, may plan major shopping trips on a preferred day and at preferred intervals [35]. Other plausible strategies could be replacing (or combining) in-store shopping with online grocery delivery or meal kits delivery, which have also been identified as ways to tackle limited time availability [36]. Progress in digital technology and the expansion of grocery delivery and meal kits markets [37] provide new opportunities for workers to manage food-related time scarcity. Future research should explore how workers adapt their food practices to cope with food-related time scarcity in the context of the emerging food delivery system.

Although work arrangements were not associated with meal delivery frequency, longer work hours were linked to ordering in to support work tasks. For workers with longer hours, meal delivery may primarily function as a means to maintain productivity and manage work demands rather than ease household tasks or free time for leisure or healthy activities. This may reflect work intensification, where workloads are rising and pushing workers to reorganize their personal time around paid employment [38]. Recent qualitative research also shows that meal delivery users reallocate delivery waiting time to work, e.g. when having to meet deadlines [39]. Time saved on food-related tasks is thus not spent on personal time but redirected towards meeting work demands. This pattern is consistent with the conservation of resources theory and the job demands-resources model [22, 23], where individuals engage in behavioural strategies to conserve limited time and energy resources under conditions of high work demands. In this context, meal delivery use can be understood as a strategy to save resources in one domain (e.g. cooking) and reallocate it to another (i.e. paid work). The prioritization of work over cooking may have implications for diet and food culture, normalizing reliance on ready-to-eat meals to support work demands and contributing over time to a decline in cooking skills, especially amongst younger generations. From a public health perspective, work demands should not take precedence over healthy food habits. Greater focus on sustainable workloads may help workers reclaim time and energy for personal activities [3], including food-related ones. Belgium already has legal frameworks around work time and schedule (e.g. with limits on working time, mandatory rest periods, advance notice, and the right to disconnect) [40]. Nevertheless, workers with demanding work arrangements continue to experience substantial time pressures, suggesting that formal protections may not necessarily (or sufficiently) translate into reduced day-to-day time scarcity. This may, yet again, reflect differences in implementation and accessibility across sectors and occupations, particularly for workers in roles with limited flexibility. Whilst the 5-day workweek remains the standard full-time arrangement, the introduction of compressed 4-day workweeks may create different patterns of time availability and flexibility for food-related tasks. Future research could unpack the impact of such emerging work arrangements on food practices as they gain wider uptake.

Our findings showed non-regular schedules were associated with frequent meal delivery use. Such schedules have remained largely unaltered post-pandemic and are most common in sectors such as healthcare and manufacturing [24]. Previous research has already noted detrimental implications of non-regular schedules on general health outcomes and behaviours [7, 8, 24, 25]. Our findings extend this evidence to meal delivery use, further highlighting inequalities between workers with different schedule types. Workers with non-regular schedules, such as shift and night work, often need extensive time to recover from work [25]. Greater fatigue and need for recovery [25] may limit the time and energy available for food shopping and cooking. This could potentially explain more frequent reliance on meal delivery. However, amongst workers using meal delivery services, we found no association between non-regular schedules and reasons related to leisure time. It could be that instead workers with non-regular schedules face a temporal incompatibility between their time off-work and grocery store opening hours, reducing opportunities for food shopping and making delivery a convenient alternative. Irregular work patterns may also make it harder to maintain shopping and cooking routines. Previous research has shown those working non-regular schedules may struggle to keep routines such as healthy (food) habits [24, 25], with greater consumption of convenience foods [8] and more irregular meals [7]. Non-regular schedules may thus come at the cost of healthy eating, as delivery reduces control over ingredients and portion sizes, and most options available for delivery tend to be unhealthy [17]. Ensuring nutritious meals are accessible through meal delivery platforms could help workers who rely on these services maintain a healthier diet, particularly as meal delivery becomes increasingly normalized [11]. Opportunities may also exist to build on Belgium’s existing meal voucher system (a voluntary employee benefit widely used across the country) to support healthier food choices. For example, this could involve encouraging the use of meal vouchers for healthier meal delivery options or restricting their use on meal delivery platforms to meals that meet defined nutritional criteria.

The study has several strengths and limitations. Both recruitment and survey were multilingual (English, Dutch, and French), allowing us to reach a wider and more diverse population in the Flanders and Brussels regions of Belgium. The study was the first to explore work arrangements in relation to specific food-related time scarcity in Belgium. It also explored reasons for using meal delivery services beyond general time motivations, examining more specific motivations (e.g. time for leisure or healthy activities), providing more nuanced insights into why workers use these services. Reported reasons may, however, have been influenced by social desirability bias, with participants potentially endorsing reasons perceived as more socially acceptable. Residual confounding from other contextual and occupation-related factors (e.g. physical demands, commute hours, urbanicity) cannot be excluded. Further research is also needed to unpack drivers of meal delivery use amongst workers with non-regular schedules, especially distinguishing between non-regular schedule types, e.g. rotating shifts, split shifts. Whilst we were able to adjust for household composition, it remains unknown whether participants were the main person responsible for food purchasing and preparation within their household. It also remains possible that workers who rarely cook or prefer not to spend time on food-related tasks may be more likely to choose jobs with certain arrangements, self-selection into work arrangements can therefore not be ruled out entirely. Self-selection into the study based on interest in food and meal delivery can also not be excluded. Online recruitment via social media allowed to easily reach workers with diverse work arrangements, however, it should be noted that those with severe time constraints may still have been less likely to participate, potentially leading to an under-representation of this (extreme) group. However, we did not intend to make population-level inferences. Findings are only representative of the sample at hand.

## Conclusion

More demanding work arrangements (longer hours, ≥5-day workweek, non-regular schedules, more onsite days) were consistently associated with more frequent feelings of food-related time scarcity, particularly at the highest levels of time scarcity (most of the time/always). However, this did not translate into more frequent ready-to-eat meal delivery ordering, except for those working non-regular schedules. Amongst workers who did use meal delivery, those with more demanding arrangements were more likely to report relying on it specifically as a means to support their paid employment but not for time savings to help finish household chores or get more time for healthy or leisure activities. These findings suggest workers may use meal delivery as a targeted strategy to manage work demands rather than a general response to time scarcity. Reducing work demands and workloads could help workers regain time and energy for food-related activities.

## Supplementary Material

ckag144_Supplementary_Data

## Data Availability

The dataset analysed in the current study is available from the corresponding author on reasonable request.
