# Supplementary material for: Work arrangements, food-related time scarcity, and meal delivery use: a cross-sectional analysis of the 2024 Belgian Meal Delivery Survey
Source: Eur J Public Health. 2026 Jul 30;36(4):ckag144. doi: 10.1093/eurpub/ckag144 (PMC13424439; doi:10.1093/eurpub/ckag144)
Supplement: ckag144_Supplementary_Data [file ckag144_supplementary_data.pdf]

**Supplementary material**
**Supplementary file 1:** Participant flowchart
**Supplementary file 2:** Survey questions and operationalisation
**Supplementary file 3:** Descriptive statistics for the full, complete and omitted sample
**Supplementary file 4:** Predicted probabilities from the adjusted generalised ordered logit models
**Supplementary file 5:** Sub-analyses of associations with meal delivery services use only amongst workers using meal delivery services

**Supplementary file 1: Participant flowchart**

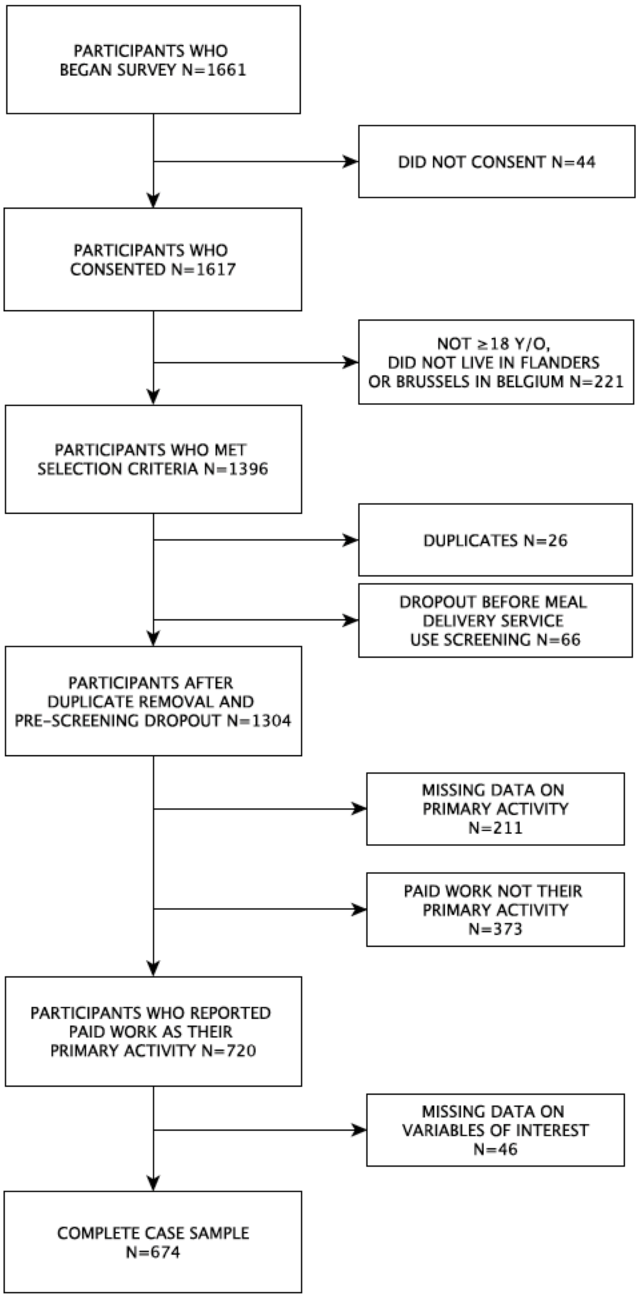

**Supplementary file 2: Survey questions and operationalisation**

| Variable | Survey questions | Response options | Derivation process | Final response |
|---|---|---|---|---|
| **Weekly work hours** | In a usual week, which of the following best describes your primary activity and/or responsibility? | 1 Employed in a paid job (including self-employed)<br>2 Homemaker or caring for family<br>3 Studying<br>4 Unemployed<br>5 Long-term sick-leave/unable to work<br>6 Retired | N/A | Hours per week |
| | [If employed]<br>In a normal week, how many hours per week do you work in your paid job(s)? Answer in hours per week. | Hours per week | | |
| **Workweek length** | [If employed]<br>In a normal week, how many days per week do you work in your paid job(s)? | 1 Less than one day per week  2 One day<br>3 Two days<br>4 Three days<br>5 Four days<br>6 Five days<br>7 Six days<br>8 Seven days | 1. Number of work days were recoded from 0 to 7, with less than one day coded 0.5.<br>2. [0.5; 4] were combined into one category capturing a workweek of less than 5 day. [5;7] were combined into another category capturing a workweek of 5 days or more | 1. <5-day workweek<br>2. ≥5-day workweek |

| | | | | |
|---|---|---|---|---|
| | | | | |
| **Work schedule** | [If employed]<br>Which category best describes your current work schedule? | 1 Regular daytime schedule<br>2 Non-daytime schedule (e.g., regular evening/night shift)<br>3 Non-regular schedule (e.g., rotating shift, split shift, on call, irregular schedule) | Categories 2 and 3 were combined to capture non-daytime, non-regular schedules. | 0 Regular daytime schedule<br>1 Non-regular schedule |
| **Number of days working onsite** | [If employed]<br>In a normal week, how many days per week do you work in your paid job(s)? | 1 Less than one day per week<br>2 One day<br>3 Two days<br>4 Three days<br>5 Four days<br>6 Five days<br>7 Six days<br>8 Seven days | 1. Number of work days were recoded from 0 to 7, with less than one day coded 0.5.<br>2. Number of working-from-home days were recoded from 0 to 7, with less than one day coded 0 (assuming people still went to work for the other half of the day)<br>3. Number of days working onsite was computed by deducting the number of working-from-home | Number of days working onsite per week |
| | [If employed]<br>In a normal week, how many days per week do you work from home? | 1 I usually do not work from home<br>2 Less than one day (e.g., only one morning or afternoon a week)<br>3 One day<br>4 Two days<br>5 Three days<br>6 Four days | | |

| | | | | |
|---|---|---|---|---|
| | | 7 Five days<br>8 Six days<br>9 Seven days per week (every day) | days from the number of total work days. | |
| **No time to shop for food** | How often do you feel that you do not have time to shop for food? | 1 Never 2 Rarely 3 Sometimes 4 Most of the time 5 Always | Categories 1 to 2 were combined into one. Categories 4 and 5 were combined into one. | 1 Never/rarely<br>2 Sometimes<br>3 Most of the time/always |
| **No time to prepare or cook a meal** | How often do you feel as though you do not have enough time to prepare and/or cook a meal? | 1 Never 2 Rarely 3 Sometimes 4 Most of the time 5 Always | Categories 1 to 2 were combined into one. Categories 4 and 5 were combined into one. | 1 Never/rarely<br>2 Sometimes<br>3 Most of the time/always |
| **Frequency of meal delivery services use** | Have you ever had a ready-to-eat meal delivered to your home? | 0 No 1 Yes | Those who never had a ready-to-eat meal delivered or not in the last 6 months were combined into one category. | 1 Never<br>2 <1/month<br>3 1-2/month<br>4 ≥3/month |
| | [if ever had a ready-to-eat meal delivered]<br>Have you had a ready-to-eat meal delivered to your home in the last 6 months? | 0 No 1 Yes | | |
| | [if had a ready-to-eat meal delivered in the last 6 months]<br>In a normal month, how often do you order a meal for delivery? | 1 <1/month<br>2 1/month<br>3 2/month<br>4 3/month<br>5 4/month<br>6 ≥5/month | Categories 2 and 3 were combined into one. Categories 4 to 6 were combined into one. | |
| **Reason for meal delivery use: easier to finish paid employment tasks** | [if had a ready-to-eat meal delivered in the last 6 months] | 0 No<br>1 Yes | N/A | 0 No<br>1 Yes |

| | | | | |
|---|---|---|---|---|
| | Based on your experiences and preferences, please indicate whether the following are reasons you order food for delivery. For each reason, choose either 'Yes, that is a reason why I order food for delivery' OR 'No, that is not a reason why I order food for delivery'.<br><br>It makes it easier for me to finish all the jobs I have to do for my paid employment or study. | | | |
| **Reason for meal delivery use: easier to finish household chores** | [if had a ready-to-eat meal delivered in the last 6 months]<br>It makes it easier for me to finish other household chores. | 0 No<br>1 Yes | N/A | 0 No<br>1 Yes |
| **Reason for meal delivery use: time for healthy activities** | [if had a ready-to-eat meal delivered in the last 6 months]<br>It gives me more free time for healthy activities such as exercise. | 0 No<br>1 Yes | N/A | 0 No<br>1 Yes |
| **Reason for meal delivery use: time for leisure activities** | [if had a ready-to-eat meal delivered in the last 6 months] | 0 No<br>1 Yes | N/A | 0 No<br>1 Yes |

| | | | | |
|---|---|---|---|---|
| | It gives me more free time for other activities such as resting, reading, watching TV, gaming. | | | |
| **Age** | What year were you born? | Year | 2024 – reported year = age | Continuous age in years |
| **Sex** | What is your gender? | 1 Man<br>2 Woman<br>3 Other | 'Other' were excluded. | 0 Man<br>1 Woman |
| **Household composition** | Do you live alone? | 0 Live with other people<br>1 Live by myself | Those reporting living alone formed one group. Those living with at least 1 adult, but no children were combined into one category. Those with children were combined into one category. | 1 Lone adult without children 2 Multiple adults without children<br>3 Household with children |
| | Not including yourself, how many people live in your household most nights of the week? | 0 0 people<br>1 1 person<br>2 2 people<br>3 3 people<br>4 4 people<br>5 5 or more people | | |
| | Children (4 years or younger) | | | |
| | Children (5 to 12 years) | | | |
| | Children (13 to 17 years) | | | |
| | Adults (18 to 25 years) | | | |
| | Adults (26 to 45 years) | | | |
| | Adults (46 to 65 years) | | | |
| | Adults (66 years or older) | | | |
| **Education** | What is the highest qualification you have completed? | 1 No diploma<br>2 Lower education<br>3 Lower secondary education of the 1st or 2nd degree | Categories 1 to 7 were combined into one category. Categories 8 and 9 were combined into another one, | 0 Non-university<br>1 University |

| | | | | |
|---|---|---|---|---|
| | | 4 Higher secondary education or secondary education of the 3rd degree<br>5 Post-secondary not-higher education (4th grade 7th year, training management  small enterprises...)<br>6 Higher education outside the university - short type, graduate (A1), professional bachelor<br>7 Higher education outside the university - the long type, master at a high school<br>8 Academic bachelor (higher school or university)<br>9 University, licentiate, engineer, master, or doctorate with thesis | | |

**Supplementary file 3: Descriptive statistics for the full, complete and omitted sample**

| | | Full sample N=720 | Complete sample N=674 | Omitted sample N=46 |
|---|---|---|---|---|
| **Weekly work hours, median** (25th perc., 75th perc.) | | 40.00 (37.50, 40.00) | 40.00 (37.50, 40.00) | 40.00 (38.00, 40.00) |
| | Missing | 18 | | |
| **Workweek length** | <5-day workweek | 104 (14.4%) | 102 (15.1%) | 2 (4.3%) |
| | ≥5-day workweek | 616 (85.6%) | 572 (84.9%) | 44 (95.7%) |
| | Missing | 0 | | |
| **Work schedule** | Regular daytime schedule | 590 (83.2%) | 568 (84.3%) | 22 (62.9%) |
| | Non-regular schedule | 119 (16.8%) | 106 (15.7%) | 13 (37.1%) |
| | Missing | 11 | | |
| **Days working onsite, median** (25th perc., 75th perc.) | | 3.00 (2.00, 5.00) | 3.00 (2.00, 5.00) | 4.00 (2.00, 5.00) |
| | Missing | 18 | | |
| **No time to shop for food** | Never/rarely | 283 (41.3%) | 274 (40.7%) | 9 (75.0%) |
| | Sometimes | 285 (41.5%) | 285 (42.3%) | 0 (0.0%) |
| | Most of the time/always | 118 (17.2%) | 115 (17.1%) | 3 (25.0%) |
| | Missing | 34 | | |
| **No time to prep/cook a meal** | Never/rarely | 220 (32.1%) | 217 (32.2%) | 3 (25.0%) |
| | Sometimes | 328 (47.8%) | 323 (47.9%) | 5 (41.7%) |
| | Most of the time/always | 138 (20.1%) | 134 (19.9%) | 4 (33.3%) |
| | Missing | 34 | | |
| **Frequency of meal delivery usage** | Never | 233 (32.4%) | 215 (31.9%) | 18 (39.1%) |
| | <1/month | 147 (20.4%) | 141 (20.9%) | 6 (13.0%) |
| | 1-2/month | 184 (25.6%) | 172 (25.5%) | 12 (26.1%) |
| | ≥3/month | 156 (21.7%) | 146 (21.7%) | 10 (21.7%) |
| | Missing | 0 | | |

| | | | | |
|---|---|---|---|---|
| **Reason for meal delivery use: easier to finish paid employment tasks** (users only) | No | 288 (59.1%) | 268 (58.4%) | 20 (71.4%) |
| | Yes | 199 (40.9%) | 191 (41.6%) | 8 (28.6%) |
| | Missing | 0 | | |
| **Reason for meal delivery use: easier to finish household chores** (users only) | No | 238 (48.9%) | 221 (48.1%) | 17 (60.7%) |
| | Yes | 249 (51.1%) | 238 (51.9%) | 11 (39.3%) |
| | Missing | 0 | | |
| **Reason for meal delivery use: time for healthy activities** (users only) | No | 414 (85.0%) | 387 (84.3%) | 27 (96.4%) |
| | Yes | 73 (15.0%) | 72 (15.7%) | 1 (3.6%) |
| | Missing | 0 | | |
| **Reason for meal delivery use: time for leisure activities** (users only) | No | 153 (31.4%) | 138 (30.1%) | 15 (53.6%) |
| | Yes | 334 (68.6%) | 321 (69.9%) | 13 (46.4%) |
| | Missing | 0 | | |
| **Age, median** (25$^{th}$ perc., 75$^{th}$ perc.) | | 34.00 (29.00, 43.00) | 34.00 (29.00, 43.00) | 34.00 (27.00, 44.00) |
| | Missing | 4 | | |
| **Sex** | Man | 311 (43.6%) | 290 (43.0%) | 21 (53.8%) |
| | Woman | 402 (56.4%) | 384 (57.0%) | 18 (46.2%) |
| | Missing | 7 | | |
| **Household composition** | Lone adult without children | 200 (27.8%) | 185 (27.4%) | 15 (32.6%) |
| | Multiple adults without children | 314 (43.6%) | 293 (43.5%) | 21 (45.7%) |
| | Household with children | 206 (28.6%) | 196 (29.1%) | 10 (21.7%) |
| | Missing | 0 | | |
| **Education** | Non-university | 265 (36.9%) | 245 (36.4%) | 20 (44.4%) |
| | University | 454 (63.1%) | 429 (63.6%) | 25 (55.6%) |
| | Missing | 1 | | |

**Supplementary file 4: Predicted probabilities from the adjusted generalised ordered logit models**

**Predicted probabilities from the work hours models**

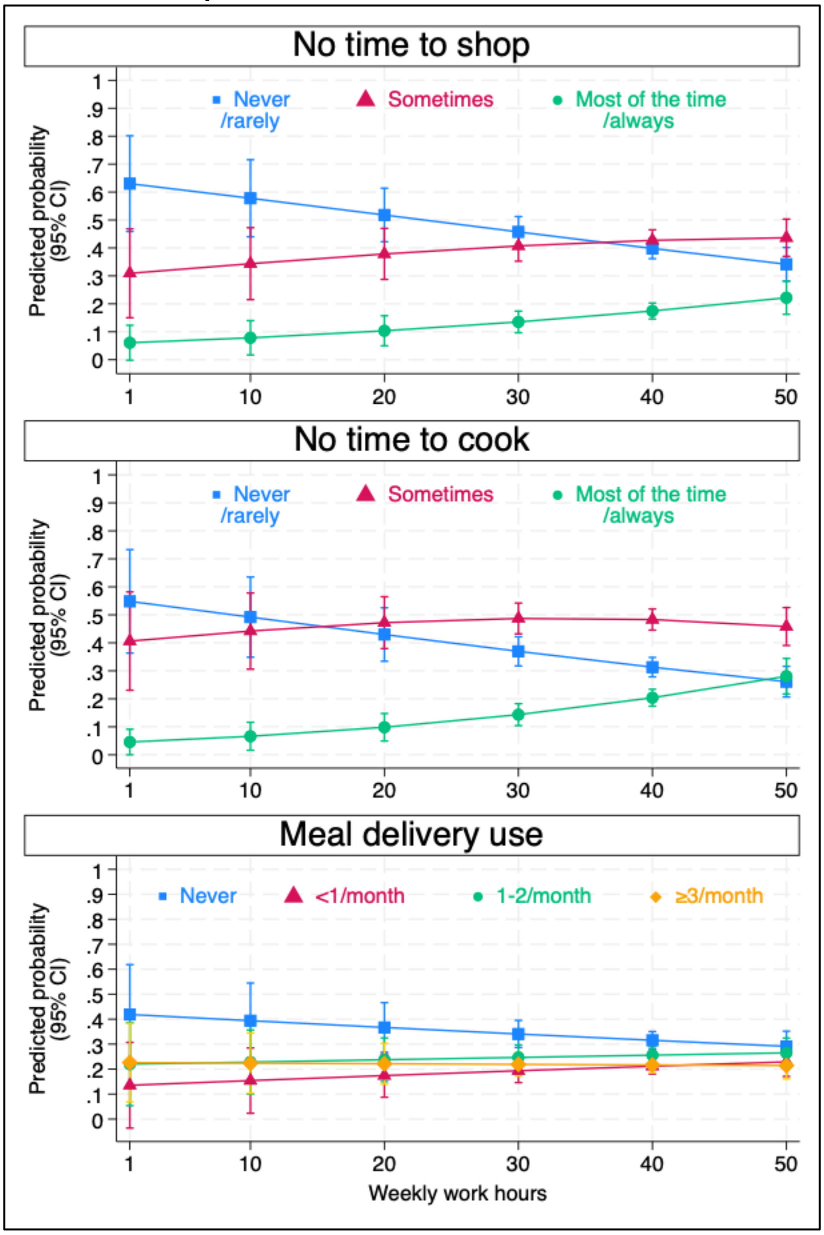

**Predicted probabilities from the workweek length models**

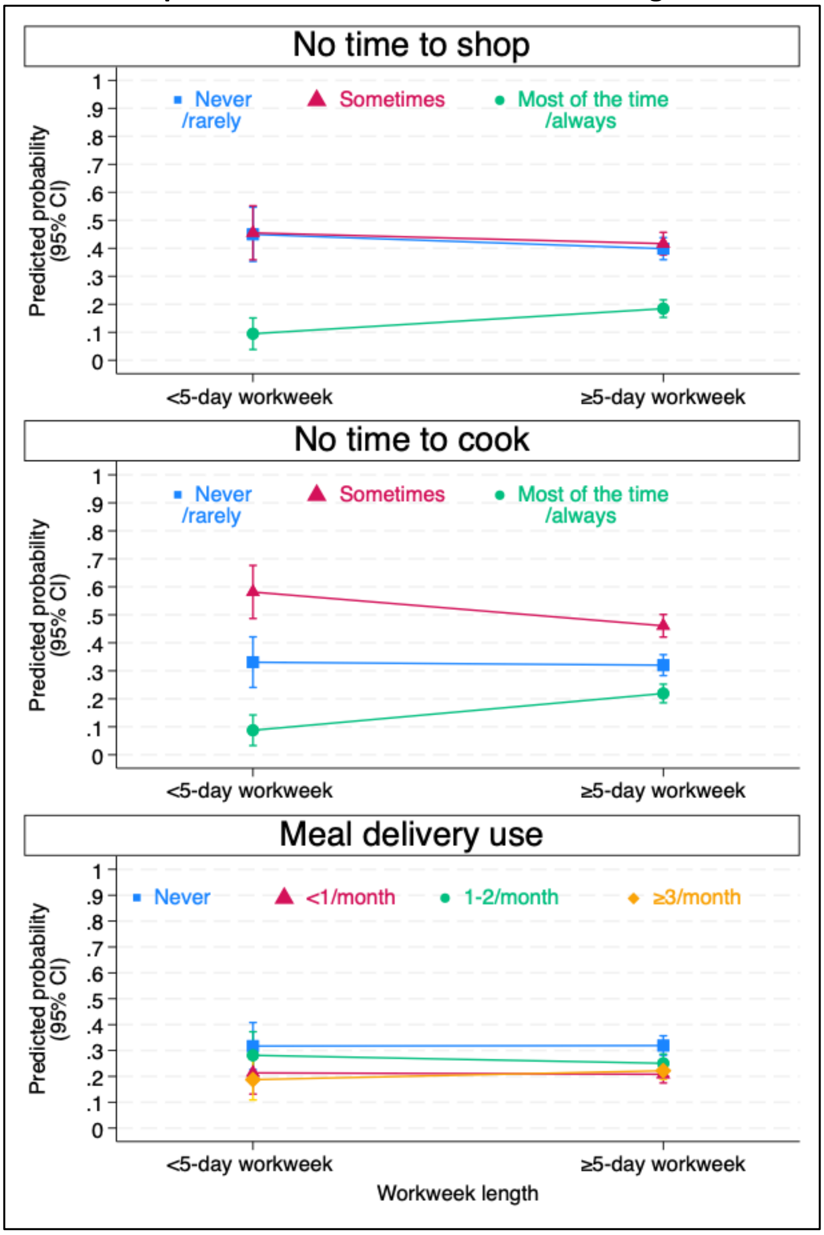

**Predicted probabilities from the work schedule models**

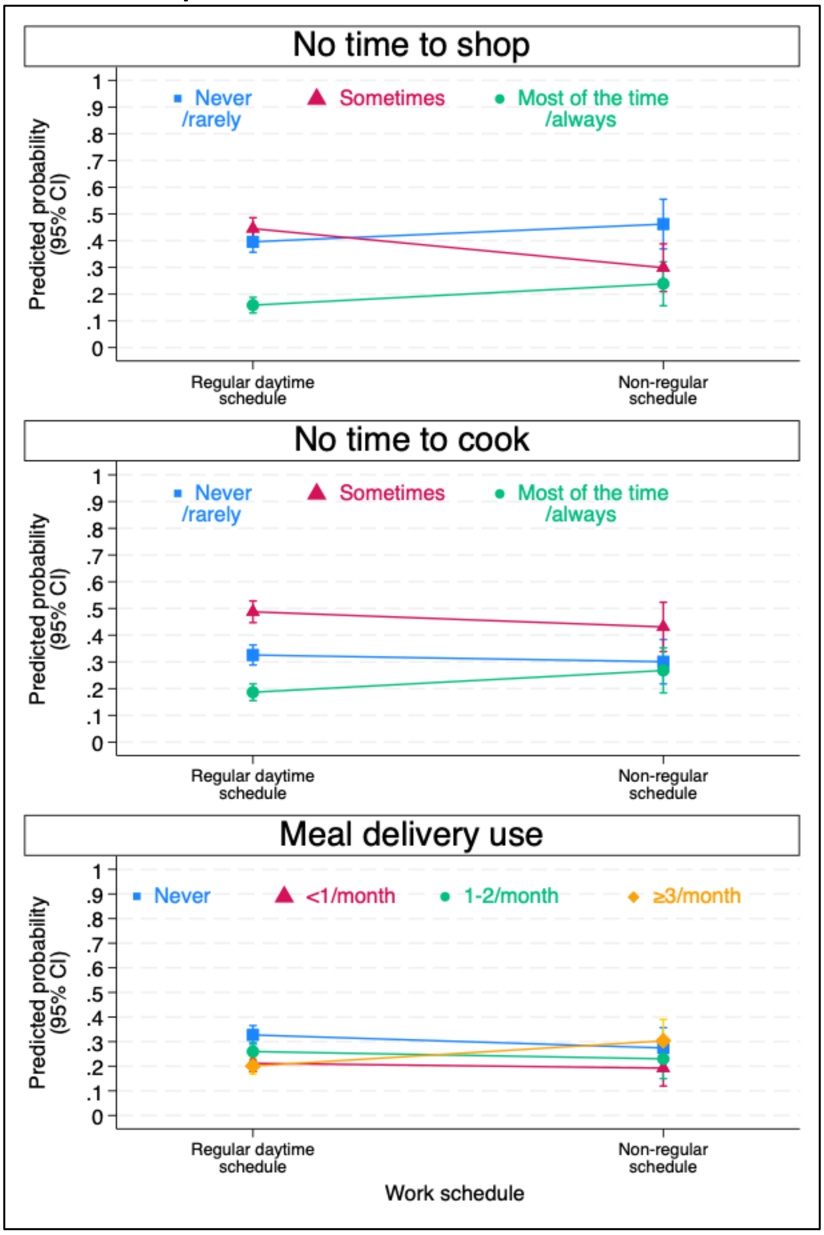

**Predicted probabilities from the days working onsite models**

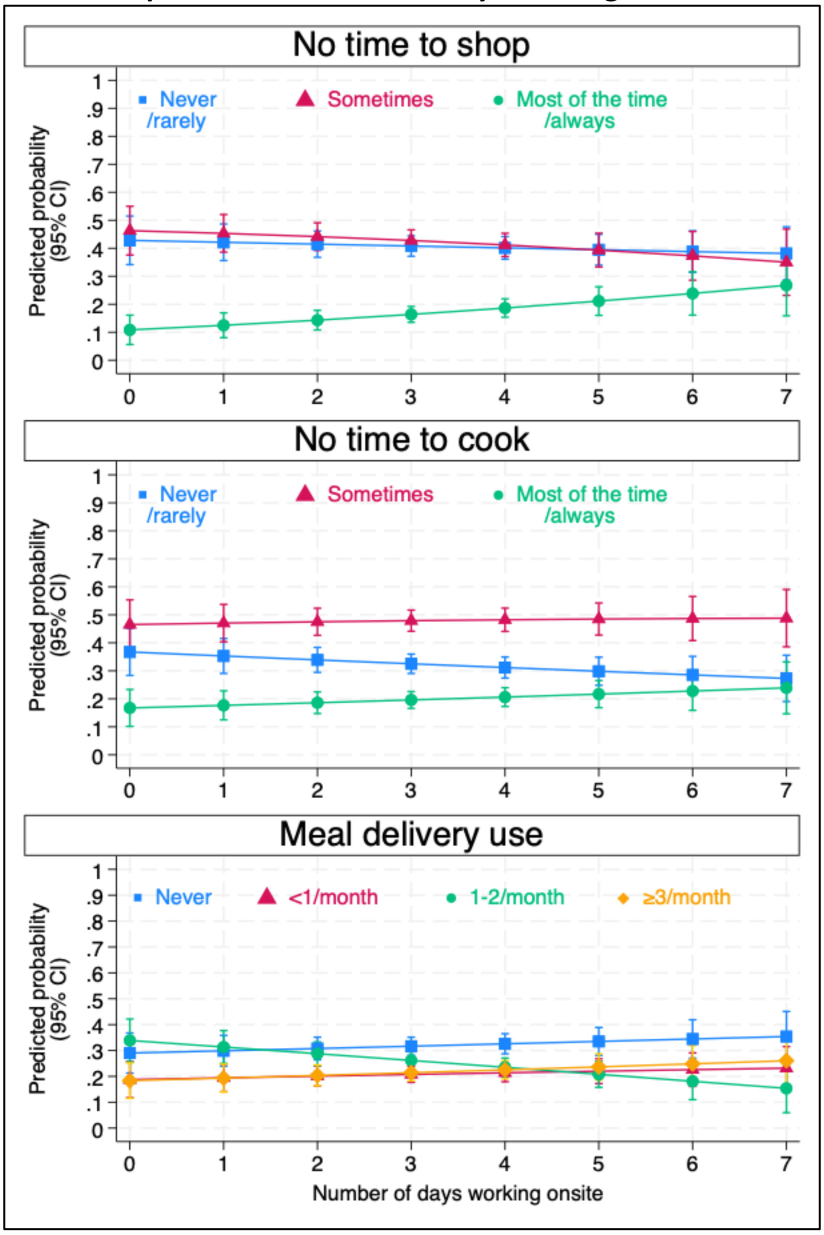

**Supplementary file 5: Sub-analyses of associations with meal delivery services use only amongst workers using meal delivery services**

These are results from separate generalised ordered logit models of the associations between work arrangements and the use of ready-to-eat meal delivery only amongst workers using meal delivery services (n=459).

| | **Lowest outcome threshold:** <1/month vs 1-2/month, ≥3/month | | **Highest outcome threshold:** <1/month, 1-2/month vs ≥3/month | |
|---|---|---|---|---|
| | **OR (95% CI)** | **p-value** | **OR (95% CI)** | **p-value** |
| **Work hours (continuous, for each 1-hour increase)** | | | | |
| Meal delivery use | 0.99 (0.97; 1.02) | 0.564 | 0.99 (0.968; 1.02) | 0.607 |
| **≥5-day workweek (ref. <5-day workweek)** | | | | |
| Meal delivery use | 0.97 (0.54; 1.76) | 0.927 | 1.24 (0.68; 2.27) | 0.477 |
| **Non-regular work schedule (ref. regular daytime schedule)** | | | | |
| Meal delivery use | 1.33 (0.75; 2.34) | 0.327 | 1.71 (1.03; 2.86) | 0.040 |
| **Days working onsite (continuous, for each 1-day increase)** | | | | |
| Meal delivery use | 0.93 (0.81; 1.07) | 0.318 | 1.09 (0.95; 1.25) | 0.218 |

**Note:** The estimate represents the increase (or decrease) in the OR for being in a higher outcome category at each outcome threshold. For a continuous exposure, it represents change in OR for each 1-unit increase in exposure. For a binary exposure, it represents change in OR compared to the reference exposure category. All models were fitted separately and adjusted for age, sex, household composition, and education. Models of work hours and workweek length also adjusted for work schedule. Additional analyses included models also adjusting for household income, and no major differences in magnitude or direction of effects were observed (results not shown). OR = odds ratio; 95% CI = 95% confidence interval.
